# Does social desirability compromise self-reports of physical activity in web-based research?

**DOI:** 10.1186/1479-5868-8-31

**Published:** 2011-04-14

**Authors:** Rik Crutzen, Anja S Göritz

**Affiliations:** 1 CAPHRI, Maastricht University, The Netherlands; 2 Work, Industrial & Organizational Psychology, University of Würzburg, Germany

## Abstract

**Background:**

This study investigated the relation between social desirability and self-reported physical activity in web-based research.

**Findings:**

A longitudinal study (*N *= 5,495, 54% women) was conducted on a representative sample of the Dutch population using the Marlowe-Crowne Scale as social desirability measure and the short form of the International Physical Activity Questionnaire. Social desirability was not associated with self-reported physical activity (in MET-minutes/week), nor with its sub-behaviors (i.e., walking, moderate-intensity activity, vigorous-intensity activity, and sedentary behavior). Socio-demographics (i.e., age, sex, income, and education) did not moderate the effect of social desirability on self-reported physical activity and its sub-behaviors.

**Conclusions:**

This study does not throw doubt on the usefulness of the Internet as a medium to collect self-reports on physical activity.

## Findings

This study illuminates the relation between social desirability and self-reported physical activity (PA) in web-based research. Self-report measures are a common way of gathering data in research on PA, because self-reports require fewer financial and logistical resources compared to more traditional methods such as accelerometers. Furthermore, interventions are nowadays increasingly delivered through the Internet [1,2], because of accessibility, convenience, and anonymity. These interventions are routinely accompanied by measurements of PA. In view of the above, the Internet might be a useful environment to collect self-reports on PA. Some researchers, however, state that the social distance [3] and the impersonal nature of the Internet might inhibit trust development [4].

Social desirability - the tendency of respondents to distort self-reports in a favorable direction [5] - might compromise self-reports on PA. Research using latent state-trait models, which account for systematic effects of the situation in which measurement takes place, indicates that the largest proportion of variance in self-reports is attributable to differences in the social desirability trait as opposed to situation-specific conditions eliciting socially desirable self-reporting [6]. If self-reports are indeed influenced by social desirability, taking into account variance due to social desirability may remove some of the error due to the use of self-reports and therewith improve self-reports' validity [7]. Previous research found minimal evidence of an influence of social desirability on scores from two self-report measures of PA in young adults [8]. This research, however, was not web-based, thereby ignoring the social distance and impersonal nature of the Internet. Mode comparison studies (i.e., in which web-based assessment is compared with, for example, paper-and-pencil assessment [9,10] or with telephone interviewing [11]) have been conducted, but the focus of these studies was not on the influence of social desirability itself: These studies did not investigate whether differences in social desirability resulted in differences in self-reported PA. If social desirability is found out to cause distortion in web-based research, this would raise concerns about the validity of web-based research on PA. Therefore, we investigated the association between social desirability and web-based self-reported PA. We put forward the research question:

To what extent is social desirability associated with self-reported PA in web-based research?

Because of the social distance [3] and the impersonal nature of the Internet [4] and in line with previous findings on web-based research on substance use [12], we did not expect social desirability to bias web-based self-reports of PA. In addition, we investigated potential moderating effects of socio-demographics (i.e., age, sex, personal net monthly income in Euros [13], and level of education according to the definitions of Statistics Netherlands) on the effects of social desirability on self-reported PA. In line with a meta-analysis about social desirability distortion [14], we did not expect any moderating effects of socio-demographics.

A longitudinal study was conducted to investigate the relation between social desirability and self-reported PA in web-based research. Data were collected through the LISS panel (Longitudinal Internet Studies for the Social sciences; http://www.lissdata.nl). The reference population for the LISS panel is the Dutch speaking population permanently residing in the Netherlands. In co-operation with Statistics Netherlands addresses were drawn randomly from the nationwide address frame and potential panel members were contacted via post. The sample from the population registers includes individuals who do not have Internet access. These individuals were provided equipment to access the Internet via a broadband connection. Sample members with small-band Internet access were provided with broadband [15].

Social desirability measurements were obtained between May 2008 and August 2008 (T1). In total, 8,722 panel members were invited. Of those, 6,766 completed the social desirability measure (77.6%) - the Marlowe-Crowne Scale [16], which has been validated previously [17]. A high score indicates a large tendency to provide socially desirable responses.

This initial sample of 6,766 panel members was re-invited between November 2008 and December 2008 (T2) to complete the follow-up measures on PA as measured by the short form of the International Physical Activity Questionnaire (IPAQ) [18]. Of those invited, 5,495 completed the IPAQ (81.2%) and were included in the analyses (Table 1). Guidelines for data processing and analysis of the IPAQ (http://www.ipaq.ki.se/scoring.pdf) were adhered to.

**Table 1:** Sample characteristics (*N = *5,495)

Socio-demographics		
Age		M^3 ^= 47.1 (SD^3 ^= 16.0)
Sex	Female	54.0%
Personal net monthly income	(in Euros)	Median = 1,300
Level of education	Primary school	9.7%
	Intermediate secondary education (US: junior high school)	26.4%
	Higher secondary education/preparatory university education (US: senior high school)	11.0%
	Intermediate vocational education (US: junior college)	23.0%
	Higher vocational education (US: college)	22.3%
	University	7.6%

Social desirability (Marlowe-Crowne)		M = 5.9 (SD = 1.5) (Scale: 0-10)

IPAQ^1^	Walking	M = 342 (SD = 367)
	Moderate-intensity activity	M = 299 (SD = 355)
	Vigorous-intensity activity	M = 157 (SD = 267)
	Sedentary behavior	M = 2,471 (SD = 1,365)
	
	Total physical activity^2^	M = 3,579 (SD = 3,525)

^1^In minutes/week; following guidelines for data processing and analysis of the IPAQ (http://www.ipaq.ki.se/scoring.pdf)

^2^In MET-minutes/week: (3.3 * walking minutes/week) + (4.0 * moderate-intensity activity minutes/week) + (8.0 * vigorous-intensity activity minutes/week)

^3^M = Mean; SD = Standard Deviation

Retainees across the two time points did not differ in sex (χ^2^(1, *N *= 6,603) = .23, *p *= .64), income (t(6,285) = .72, *p *= .47), and education (χ^2^(5, *N *= 6,603) = 10.24, *p *= .07) from panel members who dropped-out. Those who dropped-out, however, were younger than those who completed both the Marlowe-Crowne Scale and the IPAQ (42.1 versus 47.1 years, t(6,601) = 9.16, *p *< .01).

Multiple regression analyses revealed that social desirability was not associated with total PA (in MET-minutes/week), nor with its sub-behaviors (i.e., walking, moderate-intensity activity, vigorous-intensity activity, and sedentary behavior); both in terms of significance and effect sizes [19,20] (Table 2). Variance due to age, sex, personal net monthly income, and education was taken into account by including these four variables as predictors in the models. Interaction terms between socio-demographics (i.e., age, sex, income, and education) and social desirability were left out of the final models, because none of them were statistically significant. Thus, socio-demographics did not moderate the effect of social desirability on total PA or its sub-behaviors.

**Table 2:** Effect of social desirability on self-reported physical activity

	**β**^**1**^	*p*
Walking	.00	.82
Moderate-intensity activity	.00	.99
Vigorous-intensity activity	-.03	.16
Sedentary behavior	.02	.11

Total physical activity^2^	.00	.88

^1^Variance due to age, sex, personal net monthly income, and education was taken into account.

^2^In MET-minutes/week: (3.3 * walking minutes/week) + (4.0 * moderate-intensity activity minutes/week) + (8.0 * vigorous-intensity activity minutes/week)

In line with our hypothesis, this longitudinal study revealed no significant associations between social desirability and self-reported PA in web-based research. Moreover, in agreement with a meta-analysis on social desirability distortion [14], socio-demographics did not moderate the relationship between social desirability and self-reported PA. A possible explanation for the lack of a noteworthy association between social desirability and self-reported PA is that the online setting increases respondents' perceived privacy. Therefore, social desirability might have exerted less influence in this web-based study in comparison with, for example, disclosure in front of an interviewer [21]. Another fact might have contributed to not finding an association between social desirability and self-reported PA: We examined a population-representative sample from an existing panel that was not focused on PA or health as opposed to measuring PA in the context of an intervention aimed at increasing PA. In the context of an intervention aimed at increasing PA the felt pressure to overstate one's PA is likely to be higher than in a non-intervention study, implying that social desirability might distort self-reports of PA in an intervention study after all. This is a subject for future research.

Although social desirability was not found to be related to self-reported PA in this web-based research, this does not imply that self-report measures reveal the same results as direct measures (e.g., accelerometers). A recent review found correlations between self-report and direct measures of PA to be low to moderate [22]. This being only a general caution as this work was not about the comparative validity of self-reports versus direct measures.

Three final points need to be made: (1) Social desirability bias is not the only source of measurement error. Recall error, for example, may also lead to measurement error as may question format [23]. (2) People who dropped-out of the study were younger than retainees. First, a certain level of drop-out is ubiquitous in longitudinal research, also on the web [24]. Second, the dropout in these studies seems to be innocuous, because socio-demographics did not moderate the impact of social desirability on self-reported health risk behaviors. (3) These studies failed to find meaningful associations between social desirability and self-reported PA. Because an absence of evidence of an association does not equal evidence of absence of an association, future research is not precluded from revealing such an association after all. However, the longitudinal nature of this study (e.g., measurements are unlikely to be distorted by participants' unintentional and intentional attempts at portraying themselves as consistent) and the reliance on a large and representative sample gives us confidence in the robustness of our results. This study, therefore, does not throw doubt on the usefulness of the Internet as a medium of obtaining self-reports of PA.

## Competing interests

The authors declare that they have no competing interests.

## Authors' contributions

RC designed the study and AG substantially contributed to the interpretation of the data. RC drafted the manuscript and AG substantially contributed to revising it. Both authors approved the final version of the manuscript.
